# Banana Peels: A Waste Treasure for Human Being

**DOI:** 10.1155/2022/7616452

**Published:** 2022-05-13

**Authors:** Wafaa M. Hikal, Hussein A. H. Said-Al Ahl, Amra Bratovcic, Kirill G. Tkachenko, Javad Sharifi-Rad, Miroslava Kačániová, Mohammed Elhourri, Maria Atanassova

**Affiliations:** ^1^Department of Biology, Faculty of Science, University of Tabuk, Tabuk 71421, Saudi Arabia; ^2^Environmental Parasitology Laboratory, Water Pollution Research Department, Environment and Climate Change Institute, National Research Centre (NRC), 33 El–Behouth St. Dokki, Giza 12622, Egypt; ^3^Medicinal and Aromatic Plants Research Department, Pharmaceutical and Drug Industries Research Institute, National Research Centre (NRC), 33 El–Behouth St. Dokki, Giza 12622, Egypt; ^4^Department of Physical Chemistry and Electrochemistry, Faculty of Technology, University of Tuzla, Tuzla, Bosnia and Herzegovina; ^5^V. L. Komarov Botanical Institute of the Russian Academy of Sciences, Saint Petersburg, Russia; ^6^Facultad de Medicina, Universidad del Azuay, Cuenca, Ecuador; ^7^Institute of Horticulture, Faculty of Horticulture and Landscape Engineering, Slovak University of Agriculture, Tr. A. Hlinku 2, 94976 Nitra, Slovakia; ^8^Rzeszow University, Institute of Food Technology and Nutrition, Department of Bioenergetics, Food Analysis and Microbiology, Cwiklinskiej 1, Rzeszow 35-601, Poland; ^9^Laboratory of Molecular Chemistry and Natural Substance, Moulay Ismail University, Faculty of Science, B.P. 11201 Zitoune, Meknes, Morocco; ^10^Scientific Consulting, Chemical Engineering, UCTM, Sofia, Bulgaria

## Abstract

In recent years, scientists' interest in agricultural waste has increased, and the waste has become attractive to explore and benefit from, rather than being neglected waste. Banana peels have attracted the attention of researchers due to their bioactive chemical components, so we focused on this review article on the antioxidant and antimicrobial activities of banana peels that can be used as good sources of natural antioxidants and for pharmaceutical purposes in treating various diseases. Banana is an edible fruit belonging to the genus *Musa* (Musaceae), cultivated in tropical and subtropical regions. Banana peels are used as supplementary feed for livestock in their cultivation areas. Its massive by-products are an excellent source of high-value raw materials for other industries by recycling agricultural waste. Hence, the goal is to use banana by-products in various food and nonfood applications and sources of natural bioactive compounds. It can be concluded that banana peel can be successfully used in food, pharmaceutical, and other industries. Therefore, banana residues may provide new avenues and research areas for the future.

## 1. Introduction

Banana (*Musa* spp., Musaceae family) is one of the main fruit crops cultivated for its edible fruits in tropical and subtropical regions [1]. The global production of bananas is 116 million tonnes during 2019, and the banana fruits are obtained throughout the year. The fruit average is 125 grams, of which approximately 75% is water and 25% dry matter content [1]. Banana fruits vary in size and colors when ripe, from yellow, purple, and red. However, almost all culinary bananas have fruits without seeds, although wild types have fruits with many large and hard seeds [1, 2]. The fruits are eaten raw, cooked, or dried and ground as flour and used in baking [1, 2]. Besides, unripe or green bananas are used for cooking various dishes and producing starch [1, 2]. Bananas may be easily damaged during transport to the markets, and a proportion of ripe bananas are damaged and lost [1]; banana peel and plant parts are included in animal feed [1, 2].

Dessert banana, the most common and eaten, belongs to *M. acuminata* or hybrid *Musa* x *paradisiaca* or *M. sapientum* (*M. acuminata* x *M. balbisiana*) Morton [3]. The most important banana cultivar is Cavendish, which accounts for the bulk of bananas exported from the tropics and subtropics regions. Bananas are an important source of vitamin B6, vitamin C, and potassium.

The world production of bananas is divided according to their use into two groups: (1) Bananas, whose ripe fruit is eaten as a dessert. It accounts for 56% of global banana production and 97% of exports [4–6]. (2) Bananas used in cooking include bananas and other subgroups of cultivars such as “Pisang Awak” in Asia and represent 44% of global banana production [4, 5]. The ripe fruit is eaten fresh as a dessert or baked, fried, dried, or roasted. It can also be processed into vinegar, chips, or starch. The underground stem and male flowers can be eaten as a vegetable [6]. It has been estimated that 30–40% of the total banana production is rejected due to not meeting quality standards. Green fruits are easier to decompose than ripe fruits, making them wasted fruit and available to livestock [6, 7]. The leaves are also used to wrap food for cooking, make clothes, and polish floors. Banana waste includes small-sized, damaged, or rotting fruit, banana peels, leaves, stems, and pseudoparts. Fresh bananas and dry bananas can be added with various crops and additives, including molasses, grass, legumes, and rice bran. Banana and banana leaves, whole pseudostalks, or stalks can be chopped fresh, fed directly, or sliced with molasses [8].

### 1.1. Banana Peels

Banana peel is the outer shell (cover) of the banana fruit. It is a by-product of home consumption and the processing of bananas [6]. It is used as animal food. However, there are some concerns about the effect of tannin in the husks on the animals that consume it [9, 10]. Banana peels are also used as an ingredient in cooking, water purification, the manufacture of many biochemical products, and inorganic waste production [8, 11]. Banana peels are sometimes used as feedstock for livestock, goats, monkeys, poultry, rabbits, fish, zebras, and many other species [1].

### 1.2. Nutritional Value of Banana Peel

The nutritional value of banana peels varies based on the cultivar and maturity stage, as the plantain peel contains less fiber than dessert banana peels, and lignin content increases with ripening (from 7 to 15% dry matter). Dried banana peels contain 6–9% protein and 20–30% fiber. Green plantain peels contain 40% starch that is transformed into sugars after ripening. Green banana peels contain much less starch (about 15%) than green plantain peels, while ripe banana peels contain up to 30% free sugars [9]. With the use of banana peels in water purification [12], it is used to produce ethanol [13], cellulase [14], and laccase (poly copper oxidase) [15] as a fertilizer [16] and in fertilization [17].

## 2. Chemical Composition of Banana Peel

**Figure 1:**
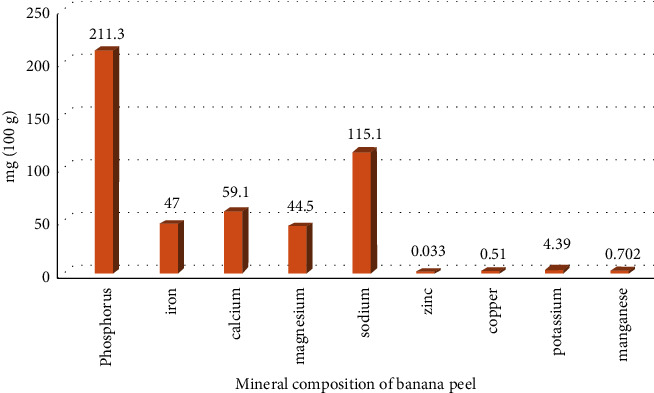
Mineral composition of banana peel [18].

It has been shown that banana peel (*Musa sapientum*) contains many nutrients and minerals [18]. They found crude proteins in the amount of 1.95 ± 0.14%, crude fat 5.93 ± 0.13%, and 11.82 ± 2.17% carbohydrate in the banana peel. The mineral composition of banana peel was phosphorus, iron, calcium, magnesium, and sodium. Zinc, copper, potassium, and manganese were found in very low concentrations as mg/100 g (Figure 1).

However, Nagarajaiah and Prakash [19] indicated in their study lower content of iron compared to the results of Hassan et al. [18]. They reported the highest amount of iron in three varieties of banana, namely, Pachabale (10 mg/100 g), Nendranbale (4 mg/100 g), and Yelakkibale (3.33 mg/100 g). The polyphenols were in the range of 200–850 mg equivalent of tannic acid/100 g. They also reported the phosphorus concentration similar to Hassan et al. 2018 [18] for Yelakkibale. However, the concentration of phosphorus was lower for both Pachabale and Nendranbale, respectively. Interestingly, they showed a very high calcium concentration (244.68/100 g) in Yelakkibale, five times higher than Hussein et al. [18] mentioned, 204.80 mg/100 g in Nendranbale and 166.54 mg/100 g in Pachabale. One more interesting detail is vitamin C, tannins, phytic acid, total oxalate, and water-soluble oxalate concentrations were significantly higher in Yelakkibale than in Nendranbale and Pachabale. Vitamin C concentration was 17.83 mg/100 g in Yelakkibale and ten times lower in both Nendranbale and Pachabale, respectively. The concentration of tannin in Yelakkibale was 1073 mg/100 g, followed by 1114 mg/100 g in Nendranbale and 517 mg/100 g in Pachabale.

**Figure 2:**
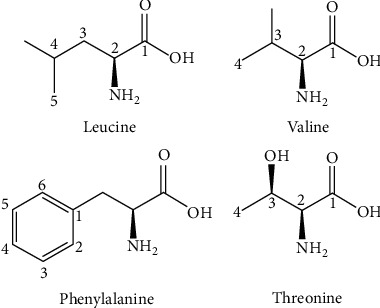
The chemical structures of some amino acids found in a banana peel: leucine, valine, phenylalanine, and threonine.

The chemical composition of six varieties of fruit peels of the banana and plantain was studied by Emaga et al. [20]. Their results reveal that the varieties did not consistently affect chemical constituents. However, the maturation of fruits involved an increase in soluble sugar content and, at the same time, a decrease in starch. The degradation of starch under endogenous enzymes may explain the increase in the soluble sugar content. They attributed the degradation of starch to the action of endogenous enzymes, which may explain the increase in the soluble sugar content. They pointed out significant quantities of amino acids such as leucine, valine, phenylalanine, and threonine. Potassium was the most important mineral element.  Figure 2 shows the chemical structures of amino acids found in a banana peel: leucine, valine, phenylalanine, and threonine.

Previous reports stated that the banana peel is rich in chemical compounds as antioxidant and antimicrobial activities. The phenolic compounds amount found in the banana peel (*Musa acuminata* Colla AAA) range from 0.9 to 3.0 g/100 g dry weight [21, 22]. Also, Someya et al. [22] identified gallocatechin at a 160 mg/100 g dry weight concentration. Ripe banana (*Musa acuminata* Colla AAA) peel also contains other compounds: anthocyanins (delphinidin and cyanidin) [23] and catecholamines [24]. On the other hand, carotenoids have been identified in the banana peel, such as *β*-carotene, *α*-carotene, and various xanthophylls, in the range of 300–400 *μ*g lutein equivalent/100 g [25], as well as sterols and triterpenes, such as *β*-sitosterol, stigmasterol, campesterol, cycloalkanol, cycloartenol, and 24-methylenecycloartanol [26].

**Figure 3:**
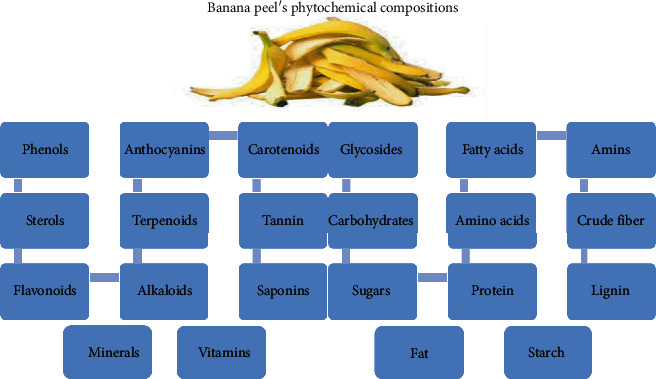
The chemical compositions of banana peels.

In 15 bananas cultivars grown in Brazil, the total phenolic content of the unripe peels ranged from 29.02 to 61.00 mg GAE/100 g and for ripe was between 60.39 and 115.70 mg GAE/100 g [27]. Also, 8 Malaysian banana cultivars showed a total phenolic content of 20.47 mg gallic acid equivalents (GAE)/100 g [28]. Mahmood et al. [29] reported that the total phenolic content was 88.31 mg tannic acid equivalent (TAE)/100 g peel (dry basis) of *M. paradisiaca*. Vipa and Chidchom [30] concluded that the tannin content was 5800 mg TAE/100 g husk (dry basis) in the ripening stage and 1130 mg TAE/100 g husk (dry basis) in the maturation stage. Also, Anal et al. [31] attained flavonoid (196 mg/g quercetin equivalent) from the banana peel extract. In the study of Behiry et al. [32], they achieved and identified rutin with a high amount (973.08 mg/100 g dry extract, *Musa paradisiaca*). Kanazawa and Sakakibara [24] reported that Cavendish banana peel extract contains naringenin, a flavanone glycoside, and a flavonol glycoside. Besides, lutein, *α*- and *β*-carotene, auroxanthin, violaxanthin, neoxanthin, *β*-cryptoxanthin, isolutein, and *α*-cryptoxanthin compounds have also been identified from banana peel extracts by Subagio et al. [25]. Plantain banana peel flour contains a total phenol level of 7.71 mg GAE/g and includes ferulic acid (0.38%) and caffeic acid (0.06%), as phenolic compounds identified in banana peel extract [33, 34], in addition to other phenolic compounds such as catecholamines and anthocyanins [35].  Figure 3 shows the chemical compounds of banana peels.

## 3. Biological Activity of Banana Peel

### 3.1. Antioxidant Activity

Several studies have proven the antioxidant activity of banana peel for its content of active compounds. Someya et al. [22] evaluated banana peel, which showed antioxidant activity due to its gallocatechin content. Ariani and Akhmad [36] explained that the antioxidant activity originated from secondary compounds in banana peels extract, such as alkaloids, flavonoids, tannins, and saponins. Flavonoids are also powerful antioxidants that can reduce free radicals [37], as free radicals damage the tissues of organs and cause various diseases. Hence, flavonoids as antioxidants are necessary to counteract the effects of free radicals in the body [36]. Another study was conducted by Mokbel and Hashinaga [38] to study the antioxidant effects of raw extracts of green banana and yellow peel. The results revealed that the water-acetone and ethyl acetate extracts of the green peel had more excellent antioxidant activity than the water-acetone and ethyl acetate extracts of the yellow peel [38]. These results agree with Jayaprakasha et al. [39] and Tepe et al. [40], and the highest efficiency was the aqueous acetone extract over all other extracts, followed by the ethyl acetate extracts. Sundaram et al. [41] reported that raw, mature, and very mature banana (*Musa paradisiaca*) peels have antioxidant activity, and raw banana peels are the most active compared to mature and very mature peels. They added that there is a positive relationship between the flavonoid content of corticosteroids and their antioxidant activity. Also, Alamsyah et al. [42] reported that banana peels (*Musa paradisiaca*) have antioxidant activity with IC50 of 64.03 ppm.

Baskar et al. [43] based their study on 9 local varieties of banana peel in Coimbatore, India. The results showed that the banana peel extract showed significant antioxidant activity. This study shows that the extract of this banana variety can be useful for treating free radical mediated diseases. Abou El-Enein et al. [44] reported that acetone extract of banana peel (*Musa paradisiaca* L.) showed the highest antimicrobial and antioxidant activities at 600 ppm, and phenolic profiles of banana peel acetone extract were chrysin, quercetin, and catechin. It was also proved by Ariani and Nurani [45] that the ethanolic extract of raw banana peel (*Musa paradisiaca* forma typical) has an extreme antioxidant activity with an IC_50_ value of 4.44 ppm. Azim et al. [46] reported that the high content of phenolic and flavonoid compounds in banana peels increases the ability to act as antioxidants and remove free radicals.

### 3.2. Antimicrobial Activity

Several works have been done to evaluate banana peel's phytochemical compositions and antimicrobial activities for using the waste for the treatment of microbial infection as possible alternatives to synthetic drugs due to those phytochemicals are safe without toxic side effects and environmental hazards [47, 48]. The results of Lino et al. [49] found that tannins present in banana peel extract have antimicrobial activity due to their astringent action, with the ability to precipitate proteins, which may affect the bacterial peptidoglycan. So, aqueous banana extracts have an inhibitory effect on Gram-positive bacteria. In the study of Mokbel and Hashinaga [38], ethyl acetate extract of green banana peel recorded significant antimicrobial activities against *Staphylococcus aureus*, *Bacillus subtilis*, *Bacillus cereus*, *Salmonella enteritidis*, and *Escherichia coli*, while yellow peel extracts recorded low activity. The data indicated that malic acid exhibited solid antibacterial activity compared to *β*-sitosterol, succinic acid, and palmitic acid; in comparison, 12-hydroxystearic acid recorded low antimicrobial activity. This study indicated that isolated compounds inhibited the growth of food poisoning bacteria in vivo [38].

Ehiowemwenguan et al. [50] studied the antibacterial activity of ethanolic extract and aqueous extract of banana peel. They concluded that ethanolic extract had the least MIC value compared to the aqueous extract. Also, they found that the organic extract of banana peel contains glycosides, alkaloids, flavonoids, and tannins. In comparison, the water extract contains only glycosides and alkaloids.

Rita et al. [51] reported that the ethanol extract of *Musa sapientum* peel inhibited 6 bacteria species. However, *Musa acuminata* peel ethanol extract has antibacterial activity against *E. coli*, *S. aureus*, and *P. aeruginosa* [52]. Wahyuni et al. [53] reported that n-butanol extract of yellow Kepok banana peels inhibited the growth of *S. aureus* and *E. coli* with MIC of 0.5 and 0.1%, respectively, and the total flavonoid and phenolic contents were 0.06 and 0.15%. Ananta et al. [54] revealed that the peels of milk, gold (lady finger), and wood banana have antibacterial activity against *E. coli* and *S. aureus*, where lady finger was the most active. Susanah et al. [55] attributed the existence of a positive correlation between the content of flavonoids or phenolic and antibacterial activities.

Several studies showed the antimicrobial activity of banana peel. Ighodaro [56] found that banana peel extract showed inhibition against *S. aureus*, *Escherichia coli*, and *Proteus mirabilis*. Also, Chabuck et al. [57] concluded that banana extract showed the highest antibacterial activity against two Gram-positive (*S. aureus* and *Streptococcus pyogenes*), four Gram-negative (*Enterobacter aerogenes*, *Klebsiella pneumoniae*, *E. coli*, and *Moraxella catarrhalis*), and one yeast (*Candida albicans*). The in vitro study of Kapadia et al. [58] found the antibacterial activity of alcoholic extract of banana peel against Gram-negative anaerobes such as *Porphyromonas gingivalis*, *Aggregatibacter actinomycetemcomitans*, and *P. gingivalis* is associated with periodontal diseases, acute periodontal abscess, and failure of the regenerative procedure [59, 60]. Also, *A. actinomycetemcomitans* is associated with aggressive periodontitis, refractory periodontitis [59, 60], and also associated with periodontitis lesion of Papillon–Lefèvre syndrome [61]. The study of Kapadia et al. [58] detected the antibacterial activity of alcoholic extract of banana peel. The results have shown a 15 mm and 12 mm inhibition zone of *P. gingivalis* and *A. actinomycetemcomitans*, respectively, due to secondary metabolites in banana peel such as flavonoids, tannins, phlobatannins, alkaloids, glycosides, and terpenoids [62, 63]. The presence of secondary metabolites might be responsible for the antibacterial activity of banana peel. Kapadia et al. [58] demonstrated that 70% isopropyl alcohol had shown 8 mm and 10 mm zones of inhibition with *P. gingivalis* and *A. actinomycetemcomitans*, respectively. In comparison, the alcohol extract of banana peel has shown 15 mm and 12 mm of inhibition zones with *P. gingivalis* and *A. actinomycetemcomitans*, respectively. In MIC, 70% isopropyl alcohol has shown the least sensitivity up to 31.25 *μ*g·mL^−1^ and 250 *μ*g·mL^−1^ against *P. gingivalis* and *A. actinomycetemcomitans*, respectively, whereas the alcoholic extract of banana peel showed sensitivity until 31.25 *μ*g·mL^−1^ against both strains. These results supported the previous studies [38, 50, 57] and indicated that banana peel extract showed sensitivity against both, but it has no antibacterial activity against *P. gingivalis* at lower concentrations. Okorondu et al. [64] showed that the methanol extract of *M. paradisiaca* peels showed greater antibacterial activity than that of ethanol, water, and chloroform extracts against the human pathogenic bacteria *Escherichia coli*, *Pseudomonas aeruginosa*, *Staphylococcus aureus*, and *Salmonella typhi.* Ighodaro [56] and McDonnell and Russell [65] also found that organic solvent had higher antibacterial activity than an aqueous solution due to isopropyl alcohol being used to dissolve more active compounds from the banana peel.

The study carried out by Singh et al. [66] represents a new approach. They studied three different colors of banana peels: red, green, and yellow against various periodontal pathogens. They found that red bananas showed a maximum zone of inhibition of 27 mm against *Planococcus citri* and 18 mm against *S. aureus*. Green banana peel showed an inhibition zone of 19 mm against *Salmonella typhi* and *Aeromonas hydrophila*. Yellow banana peel exhibited 20 mm against *A. hydrophila* followed by 13 mm against *S. aureus*. Aldean et al. [67] showed that aqueous extraction of banana peel exhibited antibacterial activity against Gram-positive and negative bacterial isolates causing gingivitis, including *Streptococcus* species.

**Figure 4:**
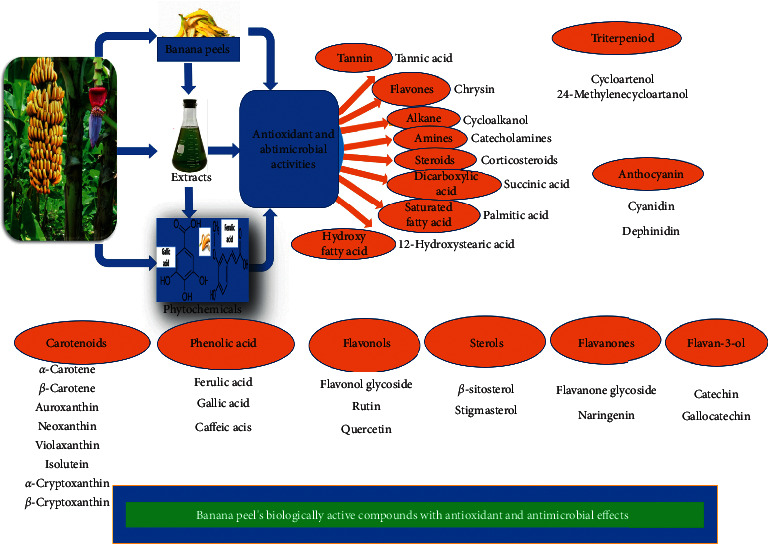
Scheme for the important banana peel's phytochemical compositions with antioxidant and antimicrobial activities.

Prakash et al. [68] showed that peel extracts from three banana varieties showed some phytochemicals, such as phenols, terpenoids, and saponins, and they exhibited antifungal activity against *A. niger*, but did not inhibit the growth of *A. flavus* or *Penicillium* spp. Also, some reports observed that gallic acid from banana peel had potential antifungal activity against four studied yeast of *Candida* spp. [69–71]. The same results were reported by Oliveira et al. [72] and Sólon et al. [73], where gallic acid had antimicrobial effects against different bacterial and fungal species. Borges et al. [74] added that ferulic acid and gallic acid had antimicrobial activity against some pathogenic bacteria. Sumathy [75] found that yellow banana fruit peel had antifungal and antimicrobial properties against different Gram-positive and negative bacteria. Lino et al. [49] concluded that banana peel inhibited the growth of enterobacteria and pyogenic bacteria. Also, Aldean et al. [67] observed that banana peel inhibited *Clostridium sporogenes*, as well as Bankar et al. [76] and Fapohunda et al. [77] noticed strong activity of banana peel extract against *K. pneumoniae*, *E. aerogenes*, and *E. coli*. Hence, Salah [78] said that bananas peel considered a good source of natural antioxidants and antibacterial, in addition to the production of natural dyes from banana peel to color cotton fabrics and protect them from bacterial effects.  Table 1 provides the biologically active compounds and especially those with antioxidant and antimicrobial effects as shown in Figure 4.

## 4. Conclusions

One of the benefits that humans get from the work of scientists on plant waste is that the banana peel was able to draw attention as a source of functional and nutritional compounds. In this work, the focus was shed on the biological activities of banana peel as antioxidant and antimicrobial activities as a result of containing biologically active compounds. Phenolic compounds, alkaloids, flavonoids, tannins, saponins, glycosides, carotenoids, sterols, triterpenes, and catecholamines isolated from banana peels have been reported for antioxidant and antimicrobial activities. It turned out that the banana peel is very encouraging for more future research. Future studies are required to determine the biologically active compounds, potentials, and the multiple benefits hoped for banana peel instead of being a neglected waste.

## Figures and Tables

**Table 1:** Banana peel's biologically active compounds with antioxidant and antimicrobial effects.

Compound	Class	References
Gallic acid	Phenolic acid	Sulaiman et al., 2011 [28]; Borges et al., 2013 [74]
Ferulic acid		Corona et al., 2015 [33]; Agama-Acevedo et al., 2016 [34]; Borges et al., 2013 [74]
Caffeic acid		Corona et al., 2015 [33]; Agama-Acevedo et al., 2016 [34]
Tannic acid	Tannin	Mahmood et al., 2011 [29]; Vipa and Chidchom, 1994 [30]
Flavanone glycoside	Flavanones	Kanazawa and Sakakibara, 2000 [24]
Naringenin		
Flavonol glycoside	Flavonols	Kanazawa and Sakakibara, 2000 [24]
Rutin		Anal et al., 2014 [31]; Behiry et al., 2019 [32]
Quercetin		Aboul-Enein et al., 2016 [44]; Ariani and Nurani, 2018 [45]
Chrysin	Flavones	Aboul-Enein et al., 2016 [44]; Ariani and Nurani, 2018 [45]
Catechin	Flavan- 3-ol	Aboul-Enein et al., 2016 [44]; Ariani and Nurani, 2018 [45]
Gallocatechin		Ariani and Akhmad, 2018 [22]; Someya et al., 2002 [36]
Lutein	Carotenoids	Subagio et al., 1996 [25]
*α*-Carotene		
*β*-Carotene		
Auroxanthin		
Neoxanthin		
Isolutein		
Violaxanthin		
*β*-Cryptoxanthin		
*α*-Cryptoxanthin		
Delphinidin	Anthocyanins	Seymour et al., 1993 [23]; González-Montelongo et al., 2010 [35]
Cyanidin		
*β*-Sitosterol	Sterols	Knapp and Nicholas, 1969 [26]
Stigmasterol		
Cycloartenol	Triterpenoid	Knapp and Nicholas, 1969 [26]
24-Methylenecycloartanol		
Cycloalkanol	Alkane	Knapp and Nicholas, 1969 [26]
Catecholamines	Amines	Kanazawa and Sakakibara, 2000 [24]; González-Montelongo et al., 2010 [35]
Corticosteroids	Steroids	Sundaram et al., 2011 [41]
Succinic acid	Dicarboxylic acid	Mokbel and Hashinaga, 2005 [38]
Palmitic acid	Saturated fatty acid	Mokbel and Hashinaga, 2005 [38]
12-Hydroxystearic acid	Hydroxy fatty acid	Mokbel and Hashinaga, 2005 [38]

## Data Availability

The data used to support this study are included within the article and are available from the corresponding author upon request.
